# Association between Dietary Niacin Intake and Migraine among American Adults: National Health and Nutrition Examination Survey

**DOI:** 10.3390/nu14153052

**Published:** 2022-07-25

**Authors:** Huanxian Liu, Lu Wang, Chunfu Chen, Zhao Dong, Shengyuan Yu

**Affiliations:** 1 Department of Neurology, Chinese PLA General Hospital, Beijing 100853, China; huanxian_liu@126.com (H.L.); yushengyuan@301hospital.com.cn (S.Y.); 2 Department of Health Care 9, The Second Medical Centre, Chinese PLA General Hospital, Beijing 100853, China; wanglu6wa@126.com; 3 Department of Neurology, Provincial Hospital Affiliated to Shandong First Medical University, Jinan 250021, China; 4 International Headache Center, Chinese PLA General Hospital, Beijing 100853, China

**Keywords:** migraine, niacin, L-shaped, cross-sectional study

## Abstract

Migraine is related to brain energy deficiency. Niacin is a required coenzyme in mitochondrial energy metabolism. However, the relationship between dietary niacin and migraines remains uncertain. We aimed to evaluate the relationship between dietary niacin and migraine. This study used cross-sectional data from people over 20 years old who took part in the National Health and Nutrition Examination Survey between 1999 and 2004, collecting details on their severe headaches or migraines, dietary niacin intake, and several other essential variables. There were 10,246 participants, with 20.1% (2064/10,246) who experienced migraines. Compared with individuals with lower niacin consumption Q1 (≤12.3 mg/day), the adjusted OR values for dietary niacin intake and migraine in Q2 (12.4–18.3 mg/day), Q3 (18.4–26.2 mg/day), and Q4 (≥26.3 mg/day) were 0.83 (95% CI: 0.72–0.97, *p* = 0.019), 0.74 (95% CI: 0.63–0.87, *p* < 0.001), and 0.72 (95% CI: 0.58–0.88, *p* = 0.001), respectively. The association between dietary niacin intake and migraine exhibited an L-shaped curve (nonlinear, *p* = 0.011). The OR of developing migraine was 0.975 (95% CI: 0.956–0.994, *p* = 0.011) in participants with niacin intake < 21.0 mg/day. The link between dietary niacin intake and migraine in US adults is L-shaped, with an inflection point of roughly 21.0 mg/day.

## 1. Introduction

Migraine is a widespread neurological condition that affects more than 1 billion people worldwide and accounts for 45.1 million years of life lived with disability [1]. According to the 2016 Global Burden of Disease, Injury, and Risk Factors Studies, migraine is the second most significant cause of disability [2], especially in those under 50 years old [3]. A recent review demonstrated that migraine is associated with nutrients [4], which can trigger migraine attacks [5,6] or reduce the prevalence of migraine [7,8]. Therefore, exploring other potential diet nutrition associated with migraine is essential, which may aid in preventing or treating it.

Niacin is a nutritional precursor for nicotinamide adenine dinucleotide (NAD) and nicotinamide adenine dinucleotide phosphate (NADP), which are required cofactors for mitochondrial energy metabolism [9]. A deficit in dietary niacin may reduce oxidative phosphorylation and disrupt mitochondrial respiration [10]. According to previous clinical research, consuming niacin orally or by injection can reduce the frequency of migraine attacks [11,12]. A previous study revealed that the trinity of brain energy deficiency, mitochondrial dysfunction, and oxidative stress might play a role in migraine development [13]. However, no research has examined the relationship between dietary niacin and migraine among the general population.

The association between dietary niacin consumption and migraine in adults was evaluated with data from the National Health and Nutrition Examination Survey (NHANES) to fill this knowledge gap. Based on the nutritional patterns found in this population, we hypothesized that individuals with migraine have a lower dietary niacin intake. In addition, the dose–response relationship between dietary niacin consumption and migraine was also assessed.

## 2. Materials and Methods

This cross-sectional study used NHANES data from 1999 to 2004, performed by the Centers for Disease Control and Prevention [14]. The objective of the NHANES project was to evaluate the health and nutritional status of non-institutionalized Americans using a stratified multistage probability survey [15]. The NHANES collects demographic and in-depth health information via home visits, screening, and laboratory testing conducted by a mobile examination center (MEC). The NHANES was authorized by the National Center for Health Statistics (NCHS) Ethics Review Committee, and all participants completed written informed consent forms before participation. The secondary analysis did not require additional Institutional Review Board approval [16]. The NHANES data are available via the NHANES website (http://www.cdc.gov/nchs/nhanes.htm) (accessed on 1 March 2022). Individuals over 20 years old who had completed an interview participated in our study. We excluded pregnant women or individuals with missing data on severe headaches or migraine, dietary niacin intake, or covariates. 

We determined whether a participant experienced migraine based on their replies to the question in the portion of the miscellaneous pain questionnaire: “Have you had a severe headache or migraine in the past three months”? In the NHANES dietary survey, respondents were questioned about the types and quantities of foods and beverages they consumed within 24 h. Dietary intake data were gathered from 1999 to 2001 using the NHANES Computer-Assisted Dietary Interview System (CADI), a multiple pass recall method that gives interviewers instructions for collecting food information. The United States Department of Agriculture collected dietary consumption data using the Automated Multiple Pass Method (AMPM) between 2002 and 2004. This fully computerized recall system comprehensively composes food-specific standard questions and possible responses. Using CADI and AMPM, accurate nutritional values were calculated for each individual depending on their consumption of food and beverages [17]. The NHANES Dietary Interviewers Procedure Manuals contains a complete overview of the dietary survey methodology [18]. The subjects were placed into four groups based on their dietary niacin intake.

A variety of potential covariates were assessed according to the literature [7,8,19,20,21], including age; sex; marital status; race/ethnicity; education level; family income; smoking status; physical activity; hypertension; diabetes; stroke; coronary heart disease; body mass index (BMI); calorie consumption; protein consumption; carbohydrate consumption; fat consumption; dietary supplements taken; and C-reactive protein. Race/ethnicity was categorized as non-Hispanic white, non-Hispanic black, Mexican American, or other races. Marital status was classified as married, living with a partner, or living alone. Educational attainment was categorized as less than 9 years, 9 to 12 years, and more than 12 years. According to a US government report [22], family income was categorized into three groups by the poverty income ratio (PIR): low (PIR ≤ 1.3), medium (PIR > 1.3 to 3.5), and high (PIR > 3.5). According to preceding literature definitions, smoking status was categorized as never smokers (smoked less than 100 cigarettes), current smokers, and former smokers (quit smoking after smoking more than 100 cigarettes). Physical activity was classified as sedentary, moderate (at least 10 min of movement within the last 30 days, resulting in only light sweating or a mild to moderate increase in breathing or heart rate), and vigorous (at least 10 min of activity within the last 30 days, resulting in profuse sweating or an increase in breathing or heart rate). The determination of previous disease (hypertension, diabetes, stroke, and coronary heart disease) was based on the inquiry in the questionnaire of whether the doctor had been informed of the condition in the past. BMI was computed using a standardized technique based on weight and height. A dietary recall interview preceded an interview at MEC to obtain participants’ 24-h nutritional information, including total dietary calories, protein, carbohydrates, and fat. Dietary supplements were determined by the question regarding nutritional supplements and medications consumed during the past month. C-reactive protein was quantified by latex-enhanced nephelometry.

This is a secondary examination of publicly accessible datasets. Categorical variables were represented by proportions (%) while continuous variables were described by the mean (standard deviation, SD) or median (interquartile range, IQR), as appropriate. To compare the differences across groups, one-way analyses of variance (normal distribution), Kruskal–Wallis tests (skewed distribution), and chi-square tests (categorical variables) were undertaken. Logistic regression models were used to determine the odds ratios (OR) and 95 percent confidence intervals (95% CIs) for the relationship between dietary niacin consumption and migraine. Model 1 was adjusted for sociodemographic characteristics, including age, sex, race/ethnicity, marital status, education level, and family income. Model 2 was adjusted for sociodemographic characteristics and the factors that *p* values were less than 0.05 in the univariate analysis. Model 3 was fully adjusted, including sociodemographic characteristics, smoking status, physical activity, hypertension, diabetes, stroke, coronary heart disease, BMI, calorie consumption, protein consumption, carbohydrate consumption, fat consumption, dietary supplements, and C-reactive protein.

In addition, restricted cubic spline (RCS) regression was performed with 4 knots at the 5th, 35th, 65th, and 95th percentiles of dietary niacin consumption to assess linearity and examine the dose–response curve between dietary niacin consumption and migraine after adjusting variables in Model 3. 

We used a two-piece-wise logistic regression model with smoothing to analyze the association threshold between dietary niacin intake and migraine after adjusting the variables in Model 3. The likelihood-ratio test and the bootstrap resampling method were used to determine inflection points.

Furthermore, potential modifications of the relationship between dietary niacin and migraine were assessed, including the following variables: sex, age (20–50 vs. >50 years), marital status (married or living with a partner vs. living alone), education level (≤12 years vs. >12 years), family income (low vs. medium or high), and BMI (<25 vs. ≥25 Kg/m^2^). Heterogeneity among subgroups was assessed by multivariate logistic regression, and interactions between subgroups and dietary niacin intake were examined by likelihood ratio testing. To evaluate the robustness of our results, we excluded participants with extreme energy intake, consuming <500 or >5000 kcal per day, for sensitivity analyses.

Because the sample size was determined solely on the data provided, no a priori statistical power estimates were performed. All analyses were performed using the statistical software packages R 3.3.2 (http://www.R-project.org, The R Foundation, Shanghai, China) (accessed on 10 March 2022). and Free Statistics software version 1.5 [23]. A descriptive study was conducted on all participants. By a two-tailed testing, a *p*-value of <0.05 was declared significant.

## 3. Results

### 3.1. Study Population

**Figure 1:**
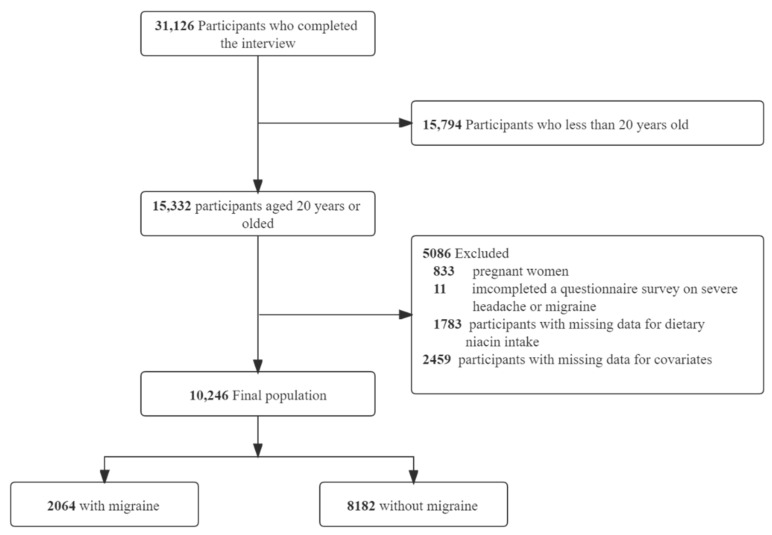
The study’s flow diagram.

In total, 31,126 participants completed the interview, of whom 15,794 participants were less than 20 years old. We excluded pregnant women (*n* = 833), those missing data on migraine (*n* = 11), those missing data on dietary niacin intake (*n* = 1783), or those with covariates (*n* = 2459). Ultimately, this cross-sectional study included 10,246 participants from the NHANES between 1999 and 2004 in the analysis. The detailed inclusion and exclusion process is shown in Figure 1.

### 3.2. Baseline Characteristics

The basic characteristics of the excluded and included participants are shown in the Supplementary Materials (Supplementary Table S1). Table 1 illustrates the baseline characteristics of all subjects according to their niacin intake quartiles. There were 2064 (20.1%) individuals with migraine. The average age of the study participants was 50.5 (18.5) years, and 5087 (49.6%) individuals were female. Individuals who consumed more niacin often tended to be younger; men; married or living with a partner; non-Hispanic white; never smokers; had a higher educational level; had a high family income; had greater physical activity; had a lower incidence of hypertension, diabetes, and stroke; and had higher consumption of calories, proteins, carbohydrates, and fats.

### 3.3. Relationship between Dietary Niacin Intake and Migraine

The univariate analysis demonstrated that age, sex, marital status, race, smoking status, family income, physical activity, coronary heart disease, BMI, protein consumption, and dietary supplements were associated with migraines (Table 2).

**Figure 2:**
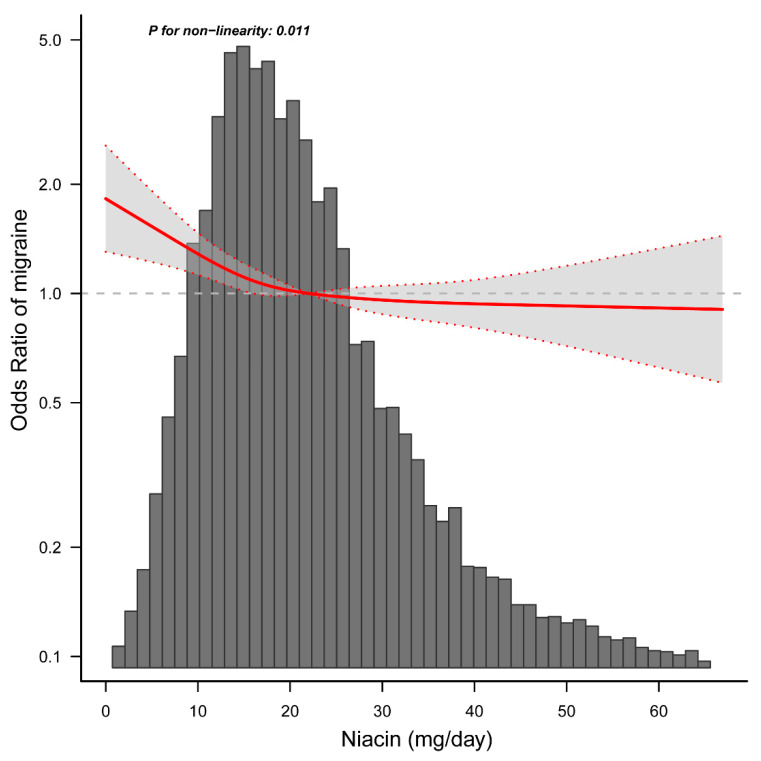
Association between dietary niacin intake and migraine odds ratio. Solid and dashed lines represent the predicted value and 95% confidence intervals. They were adjusted for age, sex, marital status, race/ethnicity, education level, family income, smoking status, physical activity, hypertension, diabetes, stroke, coronary heart disease, body mass index, energy consumption, protein consumption, carbohydrate consumption, fat consumption, dietary supplements taken, and C-reactive protein. Only 99% of the data is shown.

When dietary niacin consumption was analyzed using quartiles, there was a significant inverse association between dietary niacin consumption and migraine after adjusting for potential confounders. Compared with individuals with lower niacin consumption Q1 (≤12.3 mg/day), the adjusted OR values for dietary niacin intake and migraine in Q2 (12.4–18.3 mg/day), Q3 (18.4–26.2 mg/day), and Q4 (≥26.3 mg/day) were 0.83 (95% CI: 0.72–0.97, *p* = 0.019), 0.74 (95% CI: 0.63–0.87, *p* < 0.001), and 0.72 (95% CI: 0.58–0.88, *p* = 0.001) (Table 3), respectively. Accordingly, the association between dietary niacin intake and migraine exhibited an L-shaped curve (nonlinear, *p* = 0.011) in RCS (Figure 2). In the threshold analysis, the OR of developing migraine was 0.975 (95% CI: 0.956–0.994, *p* = 0.011) in participants with a niacin intake of <21.0 mg/day (Table 4). This means that the risk of migraine is reduced by 2.5% with every 1 mg increase in daily dietary niacin consumption. There was no association between dietary niacin consumption and migraine when the daily niacin intake was ≥21.0 mg/day (Table 4). This means that the risk of migraine no longer decreases with increasing dietary niacin intake.

### 3.4. Stratified Analyses Based on Additional Variables

**Figure 3:**
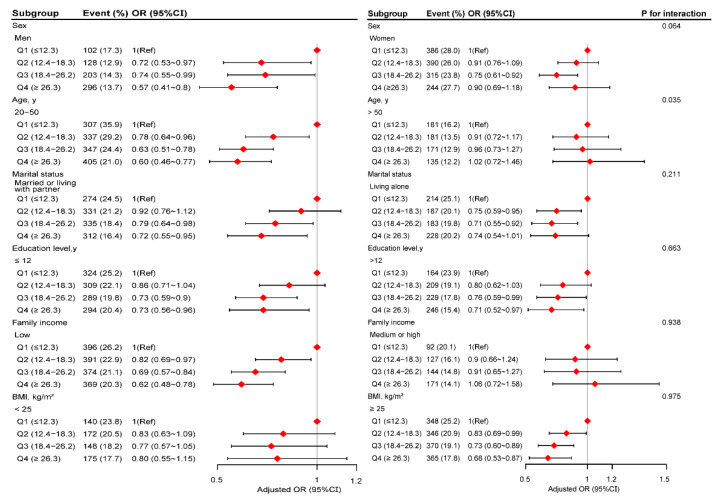
The relationship between dietary niacin intake and migraine according to basic features. Except for the stratification component itself, each stratification factor was adjusted for all other variables (age, sex, marital status, race/ethnicity, education level, family income, smoking status, physical activity, hypertension, diabetes, stroke, coronary heart disease, body mass index, energy consumption, protein consumption, carbohydrate consumption, fat consumption, dietary supplements taken, and C-reactive protein).

In several subgroups, stratified analysis was performed to assess potential effect modifications on the relationship between dietary niacin and migraine. No significant interactions were found in any subgroups after stratifying by sex, marital status, education level, family income, and BMI (Figure 3). Considering multiple testing, a *p* value of less than 0.05 for the interaction of age may not be statistically significant.

### 3.5. Sensitivity Analysis

After excluding the individuals with extreme energy intake, 9980 individuals left, and the association between dietary niacin intake and migraine remained stable. Compared with individuals with lower niacin consumption Q1 (≤12.3 mg/day), the adjusted OR values for dietary niacin intake and migraine in Q3 (18.4–26.2 mg/day) and Q4 (≥26.3 mg/day) were 0.78 (95% CI: 0.66–0.92, *p* = 0.004), and 0.74 (95% CI: 0.60–0.92, *p* = 0.006) (Supplementary Table S2), respectively.

## 4. Discussion

This large cross-sectional study of American adults demonstrated an L-shaped relationship between dietary niacin consumption and migraine, with an inflection point of almost 21.0 mg per day. Both the stratified and sensitivity analyses showed that the relationship between dietary niacin intake and migraine remained robust. 

Niacin’s influence on migraines has only been documented in a few cases. Gedye treated 12 migraine patients with 5 medicines, including niacin, and observed that 75% (9/12) of the patients benefited significantly, suggesting that niacin may be effective as an adjuvant treatment for acute migraine [24]. David et al. described a patient with migraine who experienced continuous headache relief after taking niacin with sustained release [11]. According to a literature review by Prousky et al., niacin might benefit migraine [12]. It is noteworthy that all of the studies above are case reports or case series, and no additional research has been undertaken to investigate the association between dietary niacin and migraine in the general population. The NHANES affords us the unique chance to assess whether there is an association between dietary niacin and migraine, and the dose–response link between the two, fully adjusted for numerous covariates and a range of stratified analyses. 

The relationship between dietary niacin consumption and migraine was L-shaped. The beneficial effect of increasing dietary niacin consumption on migraine seemed to peak in persons with adequate niacin intake levels. Specifically, the risk of migraine decreased with increasing dietary niacin consumption in those with a dietary niacin intake of <21.0 mg/day, whereas the risk of migraine no longer dropped with increasing dietary niacin intake in those with a dietary niacin intake of ≥21.0 mg/day. Foods rich in niacin included fish, meat, milk, peanuts, and enriched flour products [9]. From the current statical data analysis, it seems that a balanced diet helps prevent migraine. For example, the original Mediterranean diet might be rich in niacin since it contains high amounts of healthy foods, including legumes, fruits, vegetables, whole grains, olive oil, nuts, and relatively large amounts of seafood and fish, and moderate amounts of wine [25]. Moreover, a recent cross-sectional study by Arab et al. indicated that adherence to the Mediterranean diet pattern is associated with a lower migraine frequency, duration, and migraine-related disability [26]. However, the American diet, also known as the Western diet, is characterized by a richness in animal protein, refined carbohydrates, and an increased proportion of omega (ω)-6: ω-3 polyunsaturated fatty acids (PUFAs) [27]. The previous study by Sanders et al. demonstrated that omega-3 PUFAs may prevent migraine [28]. In addition, a literature review by Jahromi et al. considered that ketogenic (low-carbohydrate) and low-calorie diets may be effective strategies for migraine prevention [29]. Therefore, we speculate that the American diet may contribute to the increased prevalence of migraine.

Although the underlying mechanism of the inverse association between niacin intake and migraine is still to be investigated, our findings are biologically plausible based on the available evidence. First, prior studies demonstrated decreased platelet serotonin levels and increased urinary 5-hydroxyindoleacetic acid excretion (its principal metabolite) during migraine attacks. It is hypothesized that low serotonin levels in the systemic and central nervous systems are closely related to migraine pathogenesis [30]. Moreover, serotonin is an important neurotransmitter engaged in central antinociception. According to neuroimaging studies of migraine patients, the dorsal raphe, a major serotonin store, is thought to be implicated in the pathogenesis of migraines [31,32,33]. Niacin and its derivatives serve as negativity regulatory agencies in the kynurenine pathway, transforming the serotonin precursor tryptophan into niacin [34]. Therefore, elevated plasma niacin concentrations may shift tryptophan into the serotonin pathway, elevating plasma serotonin concentrations [24,34]. Second, a brain energy deficit was implicated in migraine pathogenesis, as evidenced by ^31^P-nuclear magnetic resonance studies [35,36,37,38,39]. Because mitochondria are energy producer factories in the brain, any mitochondrial damage can result in an energy deficiency, triggering migraine [40]. Niacin deficiency, an essential cofactor in mitochondrial oxidative phosphorylation reactions [9], will impair mitochondrial function and decrease brain energy. As a result, increasing dietary niacin intake may help improve mitochondrial function and alleviate brain energy deficiency, reducing migraine attacks to some extent. Third, oxidative stress is related to migraine development [41]. Niacin reduces oxidative stress in endothelial cells by increasing the NADP content, decreasing glutathione, and inhibiting the production of reactive oxygen species [42]. Niacin, when taken together, raises serum serotonin levels, improves brain energy deficiency, and has potent antioxidant properties, which may be the biological mechanism underlying increased niacin intake for migraine prevention. However, more prospective investigations are necessary to validate niacin’s preventive effect on migraine and its agent.

Some limitations need to be considered. First, migraine data were only collected in NHANES between 1999 and 2004. This prevented us from using NHANES data from different time periods for further validation. Second, even though regression models, stratified analyses, and sensitivity analysis were performed, residual confounding effects from unmeasured or unknown factors could not be excluded entirely. Third, the current findings were derived from a survey of adults in the United States, and whether they can be generalized to other populations requires further investigation. Fourth, participants with severe headaches or migraines were thought to have experienced migraine. This classification could not be validated against the diagnostic criteria of the International Classification of Headache Disorders (ICHD). However, according to the American Migraine Prevalence and Prevention study, 17.4% of the participants had severe headaches, with 11.8% and 4.6% meeting the ICHD 2nd edition migraine and possible migraine criteria, respectively [43]. As a result, it appears reasonable that most people who reported severe headaches in our study were considered as migraines. Of course, future studies using the ICHD 3rd edition diagnostic criteria for migraine are needed to confirm our results further. Fifth, dietary niacin intake was obtained from a 24-h recall, which may contribute to recall bias. However, the food frequency survey provides less detailed information on food types and quantities than the 24-h recall [44,45]. Finally, due to the inherent limitations of cross-sectional studies, the causal relationship between niacin and migraine cannot be determined and needs to be further confirmed by longitudinal studies in the future. In addition to the association between nutrition and migraine, we can further explore other lifestyle factors that may affect migraine in the future, such as physical activity.

## 5. Conclusions

In conclusion, there was an L-shaped connection between dietary niacin intake and migraine prevalence among adults in the United States, with an inflection point of roughly 21.0 mg/d. The results of this study draw people’s attention to the association between dietary niacin intake and migraine.

## Figures and Tables

**Table 1:** Population characteristics by categories of dietary niacin intake.

Characteristic	Niacin Intake, mg/d					
	Total	Q1 (≤12.3)	Q2 (12.4–18.3)	Q3 (18.4–26.2)	Q4 (≥26.3)	*p*-Value
NO.	10,246	1971	2494	2746	3035	
Age (year), Mean (SD)	50.5 (18.5)	54.1 (18.9)	53.0 (18.4)	50.6 (18.2)	45.9 (17.5)	<0.001
Sex, n (%)						<0.001
Male	5159 (50.4)	591 (30.0)	992 (39.8)	1422 (51.8)	2154 (71.0)	
Female	5087 (49.6)	1380 (70.0)	1502 (60.2)	1324 (48.2)	881 (29.0)	
Marital status, n (%)						<0.001
Married or living with a partner	6411 (62.6)	1120 (56.8)	1562 (62.6)	1823 (66.4)	1906 (62.8)	
Living alone	3835 (37.4)	851 (43.2)	932 (37.4)	923 (33.6)	1129 (37.2)	
Race/ethnicity, n (%)						<0.001
Non-Hispanic white	5364 (52.4)	859 (43.6)	1276 (51.2)	1498 (54.6)	1731 (57.0)	
Non-Hispanic black	1887 (18.4)	432 (21.9)	460 (18.4)	447 (16.3)	548 (18.1)	
Mexican American	2231 (21.8)	520 (26.4)	574 (23.0)	600 (21.8)	537 (17.7)	
Others	764 (7.5)	160 (8.1)	184 (7.4)	201 (7.3)	219 (7.2)	
Education level (year), n (%)						<0.001
< 9	1490 (14.5)	456 (23.1)	397 (15.9)	377 (13.7)	260 (8.6)	
9–12	4097 (40.0)	830 (42.1)	1002 (40.2)	1084 (39.5)	1181 (38.9)	
>12	4659 (45.5)	685 (34.8)	1095 (43.9)	1285 (46.8)	1594 (52.5)	
Family income, n (%)						<0.001
Low	2827 (27.6)	755 (38.3)	707 (28.3)	653 (23.8)	712 (23.5)	
Medium	3983 (38.9)	758 (38.5)	999 (40.1)	1119 (40.8)	1107 (36.5)	
High	3436 (33.5)	458 (23.2)	788 (31.6)	974 (35.5)	1216 (40.1)	
Smoking status, n (%)						<0.001
Never	5168 (50.4)	1049 (53.2)	1281 (51.4)	1392 (50.7)	1446 (47.6)	
Current	2294 (22.4)	437 (22.2)	512 (20.5)	585 (21.3)	760 (25.0)	
Former	2784 (27.2)	485 (24.6)	701 (28.1)	769 (28.0)	829 (27.3)	
Physical activity, n (%)						<0.001
Sedentary	4355 (42.5)	1061 (53.8)	1112 (44.6)	1137 (41.4)	1045 (34.4)	
Moderate	2905 (28.4)	501 (25.4)	738 (29.6)	806 (29.4)	860 (28.3)	
Vigorous	2986 (29.1)	409 (20.8)	644 (25.8)	803 (29.2)	1130 (37.2)	
Hypertension, n (%)	2778 (27.1)	619 (31.4)	747 (30.0)	772 (28.1)	640 (21.1)	<0.001
Diabetes, n (%)	1022 (10.0)	241 (12.2)	268 (10.7)	270 (9.8)	243 (8.0)	<0.001
Stroke, n (%)	333 (3.3)	88 (4.5)	101 (4.0)	79 (2.9)	65 (2.1)	<0.001
Coronary heart disease, n (%)	487 (4.8)	103 (5.2)	127 (5.1)	128 (4.7)	129 (4.3)	0.341
Body mass index (kg/m2), Mean (SD)	28.4 (6.2)	28.6 (6.20	28.3 (6.4)	28.5 (6.1)	28.2 (6.1)	0.080
Calorie consumption (kcal/d), Mean (SD)	2120.4 (1028.6)	1235.6 (527.7)	1750.3 (578.8)	2158.9 (687.8)	2964.4 (1163.3)	<0.001
Protein consumption (g/d), Mean (SD)	79.6 (42.0)	40.1 (18.0)	61.6 (19.4)	80.8 (23.6)	119.1 (45.7)	<0.001
Carbohydrate consumption (g/d), Mean (SD)	262.2 (134.6)	166.4 (84.2)	223.4 (94.0)	268.7 (105.0)	350.4 (156.0)	<0.001
Fat consumption (g/d), Mean (SD)	79.0 (46.2)	45.0 (24.6)	66.0 (29.7)	81.0 (35.6)	109.9(55.6)	<0.001
Dietary supplements taken, n (%)	5179 (50.5)	919 (46.6)	1328 (53.2)	1413 (51.5)	1519 (50.0)	<0.001
C-reactive protein (mg/dl), Median (IQR)	0.2 (0.1, 0.5)	0.3 (0.1, 0.6)	0.2 (0.1, 0.5)	0.2 (0.1, 0.5)	0.2 (0.1, 0.4)	<0.001
migraine, n (%)	2064 (20.1)	488 (24.8)	518 (20.8)	518 (18.9)	540 (17.8)	<0.001

**Table 2:** Association of covariates and migraine risk.

Variables	OR (95% CI)	*p*-Value	Variables	OR (95% CI)	*p*-Value
Age (years)	0.98 (0.97–0.98)	<0.001	Physical activity, n (%)		
Sex, n (%)			Sedentary	1(reference)	
Male	1(reference)		Moderate	0.84 (0.75–0.95)	0.005
Female	2.16 (1.96–2.39)	<0.001	Vigorous	0.82 (0.73–0.92)	0.001
Marital status, n (%)			Hypertension, n (%)		
Married or living with a partner			No	1(reference)	
Living alone	1.11 (1.00–1.22)	0.045	Yes	0.96 (0.87–1.08)	0.520
Race/ethnicity, n (%)			Diabetes, n (%)		
Non-Hispanic white	1(reference)		No	1(reference)	
Non-Hispanic black	1.30 (1.14–1.48)	<0.001	Yes	0.91 (0.78–1.08)	0.290
Mexican American	1.24 (1.09–1.40)	0.001	Stroke, n (%)		
Others	1.38 (1.15–1.66)	<0.001	No	1(reference)	
Education level (years), n (%)			Yes	1.18 (0.91–1.53)	0.216
<9	1(reference)		Coronary heart disease, n (%)		
9–12	1.13 (0.98–1.31)	0.102	No	1(reference)	
>12	0.88 (0.76–1.01)	0.075	Yes	0.64 (0.5–0.83)	0.001
Smoking status, n (%)			Body mass index (kg/m^2^)	1.02 (1.01–1.03)	<0.001
Never	1(reference)		Calorie consumption (kcal/d)	1.00 (1.00–1.00)	0.521
Current	1.29 (1.15–1.45)	<0.001	Protein consumption (g/d)	1.00 (1.00–1.00)	0.012
Former	0.69 (0.61–0.78)	<0.001	Carbohydrate consumption (g/d)	1.00 (1.00–1.00)	0.051
Family income, n (%)			Fat consumption (g/d)	1.00 (1.00–1.00)	0.600
Low	1(reference)		Niacin consumption (mg/d)	0.99 (0.99–1.00)	<0.001
Medium	0.73 (0.65–0.82)	<0.001	Dietary supplements taken, n (%)	0.80 (0.73–0.88)	<0.001
High	0.53 (0.47–0.6)	<0.001	C-reactive protein (mg/dl)	1.04 (1.00–1.09)	0.066

**Table 3:** Association between dietary niacin intake and migraine.

Quartiles	OR (95% CI)								
	No.	Crude	*p*-Value	Model 1	*p*-Value	Model 2	*p*-Value	Model 3	*p*-Value
Dietary niacin (mg/day)									
Q1 (≤12.3)	1971	1(Ref)		1(Ref)		1(Ref)		1(Ref)	
Q2 (12.4–18.3)	2494	0.80 (0.69–92)	0.002	0.86 (0.75–1.00)	0.051	0.85 (0.73–0.98)	0.030	0.83 (0.72–0.97)	0.019
Q3 (18.4–26.2)	2746	0.71 (0.61–0.81)	<0.001	0.80 (0.69–0.93)	0.003	0.76 (0.65–0.89)	0.001	0.74 (0.63–0.87)	<0.001
Q4 (≥26.3)	3035	0.66 (0.57–0.76)	<0.001	0.80 (0.68–0.93)	0.004	0.72 (0.59–0.88)	0.001	0.72 (0.58–0.88)	0.001
Trend test	10,246		<0.001		0.003		0.001		0.001

Q, quartiles; OR, odds ratio; CI, confidence interval; Ref: reference. Model 1 was adjusted for sociodemographic variables (age, sex, marital status, race/ethnicity, education level, family income). Model 2 was adjusted for sociodemographic (age, sex, marital status, race/ethnicity, education level, family income), smoking status, physical activity, body mass index, coronary heart disease, protein consumption, and dietary supplements taken. Model 3 was adjusted for sociodemographic (age, sex, marital status, race/ethnicity, education level, family income), smoking status, physical activity, hypertension, diabetes, stroke, coronary heart disease, body mass index, energy consumption, protein consumption, carbohydrate consumption, fat consumption, dietary supplements taken, and C-reactive protein.

**Table 4:** Threshold effect analysis of the relationship of niacin intake with migraine.

Niacin Intake mg/day	Adjusted Model	
	OR (95% CI)	*p*-value
<21.0	0.975 (0.956–0.994)	0.011
≥21.0	0.998 (0.987–1.009)	0.692
Log-likelihood ratio test		0.004

OR, odds ratio; CI, confidence interval. Adjusted for sociodemographic (age, sex, marital status, race/ethnicity, education level, family income), smoking status, physical activity, hypertension, diabetes, stroke, coronary heart disease, body mass index, energy consumption, protein consumption, carbohydrate consumption, fat consumption, dietary supplements taken, and C-reactive protein. Only 99% of the data is displayed.

## Data Availability

Publicly available datasets are available online for this study. The repository/repositories name and accession numbers are available online at http://www.cdc.gov/nchs/nhanes.htm (accessed on 1 March 2022).

## Supplementary Materials

The following supporting information can be downloaded at: www.mdpi.com/article/10.3390/nu14153052/s1, Table S1: Comparison of the basic characteristics between the excluded and included populations. Table S2: Association between dietary niacin intake and migraine in participants with extreme energy intake was not included.
