# Supplementary material for: Association between Dietary Niacin Intake and Migraine among American Adults: National Health and Nutrition Examination Survey
Source: Nutrients. 2022 Jul 25;14(15):3052. doi: 10.3390/nu14153052 (PMC9330821; doi:10.3390/nu14153052)
Supplement: Supplementary file 1 [file nutrients-14-03052-s001.zip › nutrients-1825035-supplementary.pdf]

Table S1. Comparison of basic characteristics between excluded and included populations.

Characteristic	Total (n = 31126)	Excluded population (n = 20880)	Included population (n = 10246)	*P*-value
Age (year), Mean (SD)	29.7 (25.0)	19.5 (21.2)	50.5 (18.5)	< 0.001
**Sex, n (%)**				< 0.001
Male	15184 (48.8)	10025 (48)	5159 (50.4)	
Female	15942 (51.2)	10855 (52)	5087 (49.6)	
**Marital status, n (%)**				< 0.001
Married or living with a partner	9201 (45.8)	2790 (28.4)	6411 (62.6)	
Living alone	10881 (54.2)	7046 (71.6)	3835 (37.4)	
**Race/ethnicity, n (%)**				< 0.001
Non-Hispanic white	12106 (38.9)	6742 (32.3)	5364 (52.4)	
Non-Hispanic black	7572 (24.3)	5685 (27.2)	1887 (18.4)	
Mexican American	8688 (27.9)	6457 (30.9)	2231 (21.8)	
Others	2760 (8.9)	1996 (9.6)	764 (7.5)	
**Education level (years), n (%)**				< 0.001
< 9	2456 (16.1)	966 (19.2)	1490 (14.5)	
9-12	6225 (40.8)	2128 (42.3)	4097 (40)	
>12	6592 (43.2)	1933 (38.5)	4659 (45.5)	
**Smoking status, n (%)**				< 0.001
Never	7905 (51.7)	2737 (54.2)	5168 (50.4)	
Current	3301 (21.6)	1007 (19.9)	2294 (22.4)	
Former	4089 (26.7)	1305 (25.8)	2784 (27.2)	
**Family income, n (%)**				< 0.001
Low	10780 (38.1)	7953 (44.1)	2827 (27.6)	
Medium	10317 (36.5)	6334 (35.2)	3983 (38.9)	
High	7165 (25.4)	3729 (20.7)	3436 (33.5)	
**Physical activity, n (%)**				< 0.001

Sedentary	8336 (37.5)	3981 (33.3)	4355 (42.5)	
Moderate	5270 (23.7)	2365 (19.8)	2905 (28.4)	
Vigorous	8600 (38.7)	5614 (46.9)	2986 (29.1)	
Hypertension, n (%)	4102 (22.1)	1324 (15.9)	2778 (27.1)	<0.001
Diabetes, n (%)	1576 (5.3)	554 (2.9)	1022 (10)	<0.001
Stroke, n (%)	606 (4.0)	273 (5.4)	333 (3.3)	<0.001
Coronary heart disease, n (%)	707 (4.6)	220 (4.4)	487 (4.8)	0.351
Body mass index (kg/m^2), Mean (SD)	24.9 (7.1)	22.6 (6.7)	28.4 (6.2)	< 0.001
Energy (kcal/d), Mean (SD)	2030.0 (1000.3)	1975.8 (978.9)	2120.4 (1028.6)	< 0.001
Protein intake (g/day), Mean (SD)	72.7 (41.0)	68.6 (39.8)	79.6 (42.0)	< 0.001
Carbohydrate intake (g/d), Mean (SD)	263.4 (135.4)	264.1 (135.9)	262.2 (134.6)	0.270
Niacin (mg/d), Mean (SD)	20.9 (12.6)	19.9 (12.3)	22.6 (13.0)	< 0.001
Fat intake (mg/d), Mean (SD)	75.0 (43.7)	72.6 (41.9)	79.0 (46.2)	< 0.001
Dietary supplement, n (%)	11603 (37.4)	6424 (30.9)	5179 (50.5)	<0.001
C-reactive protein (mg/dl), Median (IQR)	0.1 (0.0, 0.4)	0.1 (0.0, 0.2)	0.2 (0.1, 0.5)	< 0.001
migraine, n (%)	3045 (19.9)	981 (19.3)	2064 (20.1)	0.237

Table S2. Association between dietary niacin intake and migraine in participants with extreme energy intake was not included.

Quartiles	OR (95% CI)								
	No.	Crude	*P*-value	Model 1	*P*-value	Model 2	*P*-value	Model 3	*P*-value
Dietary niacin (mg/day)									
Q1 (≤ 12.3)	1865	1(Ref)		1(Ref)		1(Ref)		1(Ref)	
Q2 (12.4-18.3)	2489	0.82 (0.71~0.95)	0.008	0.89 (0.77~1.04)	0.138	0.88 (0.76~1.03)	0.115	0.87 (0.74~1.01)	0.075
Q3(18.4-26.2)	2742	0.73 (0.63~0.84)	<0.001	0.83 (0.71~0.96)	0.013	0.80 (0.68~0.95)	0.009	0.78 (0.66~0.92)	0.004
Q4 (≥ 26.3)	2884	0.66 (0.57~0.76)	<0.001	0.80 (0.69~0.94)	0.006	0.76 (0.61~0.93)	0.009	0.74 (0.60~0.92)	0.006
Trend test	9980		<0.001		0.004		0.005		0.003

Q, quartiles; OR, odds ratio; CI, confidence interval; Ref, Reference.

Model 1 was adjusted for sociodemographic (age, sex, marital status, race/ethnicity, education level, family income).

Model 2 was adjusted for sociodemographic (age, sex, marital status, race/ethnicity, education level, family income), smoking status, physical activity, body mass index, coronary heart disease, protein consumption, and dietary supplements taken.

Model 3 was adjusted for sociodemographic (age, sex, marital status, race/ethnicity, education level, family income), smoking status, physical activity, hypertension, diabetes, stroke, coronary heart disease, body mass index, energy consumption, protein consumption, carbohydrate consumption, fat consumption, dietary supplements taken, and C-reactive protein.
